# Combined effect of glycemic and blood pressure control on diabetic retinopathy among Chinese with type-2 diabetes mellitus

**DOI:** 10.1186/s13098-018-0377-7

**Published:** 2018-10-01

**Authors:** Chen-Wei Pan, Shan Wang, Cai-Lian Xu, E. Song

**Affiliations:** 1 School of Public Health, Medical College of Soochow University, Suzhou, China; 2 Jiangsu Key Laboratory of Preventive and Translational Medicine for Geriatric Diseases, Soochow University, Suzhou, China; 3 Lixiang Eye Hospital of Soochow University, 200 East Gan Jiang Road, Suzhou, 215123 China; 4 Department of Ophthalmology, The First Hospital of Jilin University, Changchun, China

**Keywords:** Diabetic retinopathy, Type-2 diabetes mellitus, Blood pressure, Glucose

## Abstract

**Background:**

To explore the associations of glycemic and blood pressure (BP) control with diabetic retinopathy (DR), with special focus on whether different combinations of categories of these two interventions are additive.

**Methods:**

A community-based survey including 913 patients with known type-2 diabetes mellitus (T2DM) was conducted in Suzhou, China. Retinal photographs were graded for the presence of DR using the Airlie House classification system. BP and blood hemoglobin A1c (HbA1C) levels were measured by standardized protocols. Binary logistic regression models were established to examine the associations of risk factors with DR.

**Results:**

The overall prevalence of any DR was 18.0% [95% confidence interval (95% CI) 15.5–20.6%] in this population. Stratified by conventional control thresholds, lower levels of either systolic blood pressure (SBP, < 140 mmHg) or HbA1C (< 7.0%) were not significantly associated with decreased susceptibility to DR, while patients simultaneously with lower HbA1C and SBP levels demonstrated 43% reduced likelihood of developing DR [adjusted odds ratio (OR) = 0.57, 95% CI 0.33–0.99, *P *= 0.045)], comparing with those with both higher levels of HbA1C (≥ 7.0%) and SBP (≥ 140 mmHg). Meanwhile, the group achieved intensive HbA1C (< 6.5%) and SBP (< 120 mmHg) control goals were found to have the smallest OR, but failed in yielding statistical significance (P = 0.10).

**Conclusions:**

In this community-based DR screening study of Chinese adults with T2DM, combination but not individual of lower SBP (< 140 mmHg) and HbA1C (< 7.0%) levels, were suggested to be associated with a significantly reduced likelihood of having DR.

## Background

It is projected that there would be more than 400 million individuals with type 2 diabetes mellitus (T2DM) worldwide by the year 2030 [1], most of whom will die or be disabled as a consequence of diabetic vascular complications [2, 3]. Diabetic retinopathy (DR) is a major microvascular complication of T2DM. Globally, DR accounts for about 5% of all blindness, affecting 2 million people throughout the world [4, 5]. In China, the population prevalence of any DR, non-proliferative DR and proliferative DR was estimated to be 1.14, 0.90 and 0.07%, respectively [6]. If no prompt action is taken, the estimated number of people affected by DR will increase from 126 million in the year 2010 and to 191 million by the year 2030 [7]. DR is the also the leading cause of blindness in working-aged adults, placing a heavy burden on society and leading to loss of human and financial resources [8–14]. Therefore it is of utmost importance to prevent this major diabetes complication from a public health perspective.

Elevated glucose and blood pressure (BP) are well-established risk factors for diabetes [15]. Both glycemia and BP lowering treatments were documented to reduce the risk of macrovascular and microvascular complications among patients with T2DM [16–19]. Nevertheless, lowering glycemia and BP in individuals with T2DM is an area of current controversy, with particular debates surrounding who should be offered therapy and what the glucose/BP targets should be achieved. When it comes to DR, the United Kingdom Prospective Diabetes Study revealed that an intensive compared with a conventional glycemia control policy could reduce the risk of DR [20], while other studies revealed inconsistent results that there was a non-significant trend toward a beneficial effect in the intensive-therapy group with respect to DRin the standard-therapy group [21–25]. A recent meta-analysis of landmark diabetes trials reported that each 10-mmHg decrease in systolic BP (SBP) was associated with a 13% reduction in the risk of DR; however, when trials were stratified by mean baseline SBP at greater than or less than 140 mmHg, the relative risk for DR was not lower in studies with greater baseline SBP [26]. Additionally, regarding therapies to lower glucose and SBP, it remains uncertain whether treatment of either alone is sufficient, or whether, to obtain maximum benefit, both of these risk factors need to be treated simultaneously [27]. Multiple risk factor intervention trials in T2DM has shown that increased benefits can be obtained by targeting several risk factors simultaneously [22, 28, 29], while other studies demonstrated that combined intensive BP and glycemic control does not produce an additive benefit on microvascular outcomes in T2DM [30, 31]. With regard to the above mentioned conflict findings, it remains unknown as to whether there is a specific threshold at which glycemic and BP control would complement each other multiplicatively in the clinical management of T2DM and DR, or if extremes of each risk factor might mitigate or enhance the benefits of controlling the other.

Diabetes is a major public health concern in the mainland of China, the world’s most populous country [32]. We undertook a cross-sectional study in urban communities located in eastern China to explore the associations of glycemic and BP control with DR, with special focus on whether different combinations of categories of these factors are additive.

## Methods

### Study design and procedure

This study was part of the Gusu Diabetic Retinopathy Screening Study on community-dwelling patients who had been previously diagnosed with T2DM in Suzhou located in eastern China. The detailed methodology and some major findings have been described in previous reports [33–35]. In Brief, all T2DM patients registered in the local Center for Disease Control and Prevention were invited to participate in the survey. The diagnosis of diabetes was based on the American Diabetes Association’s new diagnostic criterion for undiagnosed diabetes [36]. Totally, 1247 patients were in the sampling frame and 913 took part in the study, giving a response rate of 73.2%. Nonparticipants on average were younger than participants (*P *< 0.001), but there were no gender differences (*P* = 0.38).

The study adhered to the Declaration of Helsinki and ethics approval was obtained from the Institutional Review Board of the Soochow University. Written inform consent was obtained from each participant at the recruitment stage of the study.

### Diabetic retinopathy assessments

The grading of DR was performed on retinal fundus photographs. Two retinal fundus photographs centered at the optic disc and the macula were taken from both eyes using a digital retinal camera (Canon Inc., Japan). The Airlie House classification system of the Early Treatment Diabetic Retinopathy Study were used to grade retinopathy lesions [37]. Ungradable eyes were excluded from analysis.

### Measurement and definitions of covariates

Information regarding socioeconomic status and lifestyle-related factors were collected by questionnaires. Body Mass Index (BMI) was calculated as weight divided by the square of height in meters (kg/m^2^). Systolic and diastolic BP were recorded using a non-invasive BP monitor (Dinamap, Germany) by trained study nurses during the clinical examination. Non-fasting venous blood samples were collected and sent for biochemistry tests, including the analysis of hemoglobin A1c (HbA1c). Diseases histories such as nephrosis and heart disease were retrieved from health records of the participants. They were diagnosed previously by physicians and we cannot know the exact definitions as different physicians may have different diagnosis of the diseases.

### Statistical analysis

Intensive and standard glucoselowering therapy were defined as target HbA1C lower than 6.5% and 7.0%, respectively [22]. BP control were usually defined as intensive (SBP < 120 mmHg) or standard (SBP < 140 mmHg) [30]. Therefore, we divided patients into categories based on individual or combined HbA1C and SBP thresholds: HbA1C (6.5% and 7.0%) and SBP (120 and 140 mmHg), respectively.

Data were summarized using proportions, means-SD, as appropriate. Statistical analyses were performed using SPSS version 11.0 (SPSS Inc., Chicago, IL, USA). Binary logistic regression models were established to examine the associations of risk factors with DR. For multivariate analysis, only age, gender and known risk factors for diabetes such as BMI, durations of diabetes, and the presence of hyperlipidemia were adjusted in the model.

## Results

Among the 913 participants in this study, 880 had gradable retinal fundus photograph in at least one eye. Completed data of BP, HbA1C and other covariates were obtained from 719 participants, who included in the data analyses. The mean age of participants included in the analysis was 67.7 ± 8.3 years and there were more women than men (56% vs. 44%). The mean duration of diabetes was 10.5 ± 7.1 years and the mean blood level of HbA1Cwas 7.2 ± 1.3%. Table 1 demonstrates the prevalence of DR by different variables. The overall prevalence of any DR was 18.0% [95% confidence interval (95% CI) 15.5–20.6%] in this population. Older age and longer duration of diabetes was associated with a higher prevalence of DR, and higher HbA1C levels was significantly associated with an increased likelihood of having DR among T2DM patients [odds ratio (OR) = 1.22, *P *= 0.001]. However, the association between DR and SBP or DBP levels was not significant (P = 0.89 for SBP and P = 0.90 for DBP).

**Table 1:** Clinical features between participants absent and present DR

Characteristics	Absent (n = 719)	Present (n = 158)	OR (95% CI)	P*
Age	68.00 ± 8.24	66.47 ± 8.37	0.98 (0.96–1.00)	0.04
BMI	24.36 ± 3.09	23.96 ± 2.84	0.96 (0.9–1.01)	0.14
Sex				
Male	309 (43.0%)	78 (49.4%)	1.00 (ref)	
Female	410 (57.0%)	80 (50.6%)	0.77 (0.55–1.09)	0.14
Education				
High school and below	621 (86.4%)	136 (86.1%)	1.00 (ref)	
University and above	98 (13.6%)	22 (13.9%)	1.03 (0.63–1.70)	0.90
Live alone				
No	655 (91.1%)	142 (89.9%)	1.00 (ref)	
Yes	64 (8.9%)	16 (10.1%)	1.17 (0.66–2.09)	0.59
Regular tea consumption				
No	353 (49.1%)	65 (41.1%)	1.00 (ref)	
Yes	366 (50.9%)	93 (58.9%)	1.38 (0.97–1.95)	0.07
Smoking				
No	581 (80.8%)	119 (75.3%)	1.00 (ref)	
Yes	138 (19.2%)	39 (24.7%)	1.39 (0.93–2.09)	0.11
Alcohol drinking				
No	645 (89.7%)	141 (89.2%)	1.00 (ref)	
Yes	74 (10.3%)	17 (10.8%)	1.07 (0.61–1.86)	0.82
Nephrosis				
Absent	617 (87.2%)	133 (86.9%)	1.00 (ref)	
Present	92 (12.8%)	20 (13.1%)	1.09 (0.97–1.23)	0.14
Heart disease				
Absent	507 (70.5%)	113 (71.5%)	1.00 (ref)	
Present	212 (29.5%)	45 (28.5%)	0.95 (0.65–1.39)	0.79
Systolic pressure (mmHg)	142.65 ± 18.83	143.75 ± 19.22	1.00 (0.99–1.01)	0.51
Diastolic pressure (mmHg)	79.8 ± 10.43	80.2 ± 9.93	1.00 (0.99–1.02)	0.66
Diabetes duration (years)	10.3 ± 6.98	11.45 ± 7.50	1.02 (1.00–1.05)	0.07
HbA1C (%)	7.09 ± 1.27	7.49 ± 1.57	1.22 (1.08–1.38)	0.001
Hypertension				
Absent	226 (31.4%)	51 (32.3%)	1.00 (ref)	
Present	493 (68.6%)	107 (67.7%)	0.96 (0.67–1.39)	0.84
Stroke				
No	695 (96.7%)	151 (95.6%)	1.00 (ref)	
Yes	24 (3.3%)	7 (4.4%)	1.34 (0.57–3.17)	0.50
Hyperlipidemia				
Absent	517 (71.9%)	114 (72.2%)	1.00 (ref)	
Present	202 (28.1%)	44 (27.8%)	0.99 (0.67–1.45)	0.94

*DR* diabetic retinopathy

* *P* values were got from logistic regression

Participants were then stratified to categories by conventional thresholds of SBP and HbA1C therapies. As shown in Table 2, there were 46.24% of patients with SBP levels less than 140 mmHg, and 58.08% of patients with HbA1C levels less than 7.0%. In multivariate analyses, although lower levels of either SBP or HbA1C were not significantly associated with decreased risk of DR, combined lower HbA1C and blood pressure levels demonstrated a 43% reduction in the likelihood of developing DR (adjusted OR = 0.57, 95% CI 0.33–0.99, *P *= 0.045).

**Table 2:** Associations between DR and different categories of SBP and HbA1C (stratified by conversional thresholds)

Category	Without DR (n = 719)	With DR (n = 158)	OR (95% CI)*	P*
SBP (mmHg)				
≥ 140	408	87	1.00 (ref)	
< 140	311	71	1.03 (0.72–1.46)	0.89
HbA1C (%)				
≥ 7.0	326	85	1.00 (ref)	
< 7.0	393	73	0.78 (0.55–1.11)	0.17
Combined				
SBP ≥ 140 mmHg and HbA1C ≥ 7%	195	53	1.00 (ref)	
SBP < 140 mmHg or HbA1C < 7%	344	66	0.63 (0.38–1.05)	0.08
SBP < 140 mmHg and HbA1C < 7%	180	39	0.57 (0.33–0.99)	0.045

*DR* diabetic retinopathy, *SBP* systolic blood pressure, *OR* odds ratio, *95% CI* 95% confident interval

* Multivariate logistic regression models, adjusted by covariates such as gender, age, BMI, diabetes duration, and presence of hyperlipidemia

In the next step, patients were divided by intensive and standard thresholds (Table 3). Compared with levels of 6.5–6.9%, HbA1C levels lower than 6.5% did not contribute to significantly decreased risk of DR (adjusted OR = 0.87, 95% CI 0.51–1.47, *P* = 0.60). Similarly result was also observed for SBP comparison (adjusted OR = 0.71, 95% CI 0.37–1.38, *P* = 0.31; < 120 mmHg vs. 120–139 mmHg). At the same time, we also explored whether different combinations of categories of SBP and HbA1C could offer additive benefit. The group with HbA1C level of 6.5–6.9% and SBP level of 120–139 mmHg was treated as the reference group, patients with the combined lowest HbA1C (< 6.5%) and SBP (< 120 mmHg) levels yielded the lowest OR of 0.38, but this association was not statistically significant (*P* = 0.10).

**Table 3:** Associations between DR and different categories of systolic pressure and HbA1C (stratified by conversional and intensive thresholds)

HbA1C (%)	SBP (mmHg)	Without DR (n = 719)	With DR (n = 158)	OR (95% CI)*	P*
< 6.5		243	38	0.87 (0.51–1.47)	0.60
6.5–6.9		150	35	1.00 (ref)	
≥ 7		326	85	1.19 (0.74–1.93)	0.47
	< 120	75	13	0.71 (0.37–1.38)	0.31
	120–139	236	58	1.00 (ref)	
	≥ 140	408	87	0.82 (0.56–1.19)	0.30
< 6.5	< 120	37	5	0.38 (0.12–1.19)	0.10
< 6.5	139–120	75	16	0.73 (0.32–1.67)	0.46
< 6.5	≥ 140	131	17	0.58 (0.27–1.27)	0.18
6.5–6.9	< 120	20	3	0.53 (0.13–2.18)	0.38
6.5–6.9	139–120	48	15	1.00 (ref)	
6.5–6.9	≥ 140	82	17	0.52 (0.21–1.30)	0.16
≥ 7	< 120	18	5	0.87 (0.26–2.86)	0.81
≥ 7	139–120	113	27	0.74 (0.35–1.60)	0.45
≥ 7	≥ 140	195	53	0.87 (0.43–1.77)	0.70

*DR* diabetic retinopathy, *SBP* systolic blood pressure, *OR* odds ratio, *95% CI* 95% confident interval

* Multivariate logistic regression models, adjusted by covariates such as sex, age, body mass index, diabetes duration, and the presence of hyperlipidemia

## Discussion

In this community-based DR screening study of Chinese adults with T2DM living in an urban community in eastern China, we found that simultaneously lowering SBP and HbA1C levels than standard thresholds was associated with a significantly reduced likelihood of having DR. The findings suggested that combined standard therapeutic approach of simultaneously improving both blood glucose and BP might confer greater benefit than by treating either alone, which have important implications for the clinical management of DR.

To the best of knowledge, although the main effects of BP and glycemic control on the risk of diabetic vascular complications such as DR have been extensively investigated [38], the combined effect of these two interventions is less well assessed. Hypertension in type 1 diabetes patients has been shown to significantly increase the risk of proliferative DR in the Wisconsin Epidemiology Study [39]. Another study indicated that each increment of 5 mmHg in night-time systolic and diastolic blood pressure leads to an increase in about 40% in the risk of DR even in normotensive diabetic individuals [40]. Our study demonstrated that either lowering HbA1C or BP levels alone was not related to a significant reduction in the likelihood of having DR but combined lowering the levels of the two resulted in a 43% reduction in the likelihood of having DR. The magnitude of reduction reached clinical significance and might have important implications for the clinical management of patients with T2DM. Some animal models have demonstrated that combination of diabetes and hypertension lead to early and more severe markers of DR, both functional and morphological. A study on diabetic rats have observed that basement membrane thickness and permeability to serum albumin increased were significantly enhanced when diabetes coexisted with hypertension [41]. Hammes et al. have shown that the frequency of acellular capillaries, a morphological gold-standard marker of DR, was nearly twice as high in diabetic and hypertensive rates as in rats with diabetes only. In this study, hypertension-induced deposition of advanced glycation endproducts-proteins in the retinal vasculature played a central role in the acceleration of diabetic retinopathy by hypertension. Inhibition of advanced glycation endproducts formation by aminoguanidine prevented both accelerated diabetic retinopathy and thrombus formation without affecting hypertension. Therefore, the importance of hypertension in retinal disease was still debatable [42].

Both inflammation and oxidative stress could serve as the underlying mechanism for the observed association of poor BP and glycemic control with DR. Both oxidative stress [43, 44] and inflammation [45] have been strongly implicated in the pathogenesis of DR. It has been shown that hypertension could induce oxidative stress and inflammation [46], which, in turn, contribute to the development of DR. For example, in one animal study, genetic susceptibility of hypertension, before the establishment of full hypertension, was enough to lead to an earlier development of inflammation in the retina in the presence of diabetes [47]. Inflammation and oxidative stress and are closely related. On one hand, oxidative stress could result from the generation of reactive species from the inflammatory cells. On the other hand, oxidative stress may also lead to inflammation by pro-inflammatory gene expression [48]. The simultaneous presence of inflammation and oxidative stress tend to coexist in an inseparable manner in different organs, particularly in the retina [45].

Our study is one of the few studies describing the associations between DR and the combination of HbA1C and SBP levels among T2DM patients, especially in Chinese populations. We had a representative study sample and the use of standardized DR grading protocol also facilitated the comparisons among different studies. There were also some limitations of the study which should be acknowledged. First, the cross-sectional design limited our ability to determine the causal effect between exposures and outcomes. The DR status of the patients at the time of diagnosis of hypertension or diabetes or the initiation of antihypertensive or diabetic agents are not clear. It is possible that the grading of DR changes most with intensive treatment strategies. Second, information on other diabetes risk factors, such as psychological stress, unhealthy dietary practices, physical inactivity and patterns of health service utilization were not available in this study, and therefore the possibility of residual confounding in our regression analysis could not be excluded. Third, a single blood pressure measurement may not accurately indicate how blood pressure of the participants had been controlled. Fourth, lipid parameters has been reported to be associated with the presence and development of DR but were not collected in this study. Fifth, the number of participants with vision-threatening DR was small and the study had insufficient power to evaluate associations of different stages DR with glycemic and blood pressure control. Finally, it was found that patients simultaneously achieved intensive HbA1C and SBP control goals had the lowest OR without statistical significance, among different categories of combination of HbA1C and SBP levels. It may be that the overall sample size was relatively small and some categories were with quite small numbers of patients, and therefore our study may have a lack of statistical power to explore whether there was additional benefit of the combination of intensive therapies compared to standard controls. It warrants further investigation, on which field we are continuously working on.

## Conclusions

In summary, simultaneously lowering SBP and HbA1C levels than standard therapy thresholds was significantly associated with a reduced likelihood of having DR among Chinese T2DM patients. Well-designed clinical trials or longitudinal cohort studies are warranted to examine the relationship between the role of combined BP and glycemic control in the pathophysiology of DR among patients with T2DM.

## Abbreviations

T2DM: type 2 diabetes mellitus
DR: diabetic retinopathy
BP: blood pressure
CDC: Center of Disease Control and Prevention
OR: odds ratio
CI: confidence interval
